# Burdens on caregivers of patients with stroke during a pandemic: relationships with support satisfaction, psychological distress, and fear of COVID-19

**DOI:** 10.1186/s12877-022-03675-3

**Published:** 2022-12-13

**Authors:** Chieh-hsiu Liu, Yi-Jung Chen, Jung-Sheng Chen, Chia-Wei Fan, Meng-Tsang Hsieh, Chung-Ying Lin, Amir H. Pakpour

**Affiliations:** 1 Department of Family Medicine, Taoyuan General Hospital, Ministry of Health and Welfare, Taoyuan, Taiwan; 2 Institute of Allied Health Sciences, College of Medicine, National Cheng Kung University, 1 University Rd, Tainan, 701 Taiwan; 3 Department of Medical Research, E-Da Hospital, Kaohsiung, 82445 Taiwan; 4 Department of Occupational Therapy, AdventHealth University, Orlando, Florida USA; 5 Stroke Center and Department of Neurology, E-Da Hospital, Kaohsiung, 82445 Taiwan; 6 School of Medicine, College of Medicine, I-Shou University, Kaohsiung, 82445 Taiwan; 7 Institute of Clinical Medicine, College of Medicine, National Cheng Kung University, Tainan, 70101 Taiwan; 8 Department of Public Health, College of Medicine, National Cheng Kung University, Tainan, Taiwan; 9 Biostatistics Consulting Center, National Cheng Kung University Hospital, College of Medicine, National Cheng Kung University, Tainan, Taiwan; 10 Department of Occupational Therapy, College of Medicine, National Cheng Kung University, Tainan, Taiwan; 11 Department of Nursing, School of Health and Welfare, Jönköping University, Jönköping, Sweden

**Keywords:** Caregiver burden, COVID-19, Fear, Psychological distress, Stroke, Family support

## Abstract

**Background:**

Caregivers have faced unprecedented circumstances throughout the COVID-19 pandemic, but previous research only minimally addresses the caregivers' burden. Therefore, this study aimed to investigate the relationship between caregiver burden, psychological stress, satisfaction with support, and fear of COVID-19 in caregivers of patients with stroke during the pandemic.

**Methods:**

A cross-sectional survey study with total of 171 caregivers of patients with stroke in a community hospital in Taiwan. All participants completed the Zarit Burden Interview, Depression, Anxiety, Stress Scale (DASS-21), satisfaction of support survey, and Fear of COVID-19 Scale. Pearson correlations were used to examine the bivariate correlations between study variables. Then, with the control of demographic confounders, a multiple linear regression model was applied with significant variables to construct and explain caregiver burden.

**Results:**

The proposed model significantly explained the caregiver burden of caregivers of patients with stroke. Specifically, the caregiver burden was negatively correlated with satisfaction with family support, but positively with psychological distress and the fear of COVID-19.

**Conclusions:**

Caregivers of patients with stroke will suffer a greater burden if they have lower satisfaction with family support, experienced higher psychological distress, and perceived more fear of the COVID-19 pandemic. Health professionals must address these concerns, support caregivers, and enhance available resources.

## Introduction

The pandemic of coronavirus disease 2019 (COVID-19) has significantly impacted individuals and society as a whole [1–8]. Not only has the pandemic caused excess mortality [9] but also, governments across the world adopted a series of restrictive measures, such as face mask wearing, social distancing, and mobility restrictions, to contain the pandemic [10, 11]. Furthermore, the pandemic itself and related regulatory measures and adaptive behaviors to COVID-19 have substantially impacted the physical and mental health of the population across different backgrounds [12, 13]. In addition, despite some innovations in virtual care, the pandemic significantly limited access to and utilization of health and care services [14] and worsened the social inequalities in long-term care support [15].

Caregivers may be among the most vulnerable to the negative effects of the pandemic [16]. It is reported that under the challenges of the pandemic, both paid and informal caregivers (such as family members, relatives, and neighbors) including those who took care of people with dementia, with stroke, and with a variety of chronic/disabling conditions experienced lower quality of life and higher rates of negative health outcomes, such as depression, psychological stress, and anxiety [16–19]. In particular, informal caregivers who play a significant role in long-term caregiving and may not receive adequate formal support, appeared even more vulnerable during the period [20–22]. Excess workloads, constrained access to formal health and care services, and decreased social connectedness have led to a higher intensity and burden of caregiving in the time of COVID-19 [23–26].

Research has identified possible causes and risk factors for higher caregiver burdens during the COVID-19 pandemic, highlighting two main groups of variables. Caregiver burden can be defined as "the strain experienced by a person who cares for a chronically ill, disabled, or older family member" [27]. One line of literature for identifying increasing caregiver burden during the pandemic focused on the characteristics of care recipients. It is observed that deterioration of chronic medical conditions and decline in cognition and physical functions of care recipients during the pandemic have increased the burdens of caregiving [28–30]. Another line of study examined the availability of formal care resources and accessibility of care services. Some research revealed that the difficulty of utilizing formal care services before the pandemic negatively impacts caregiver burdens [31, 32]. While patients with stroke require access to rehabilitation and long-term care services, the restrictions of formal care support during the pandemic have not only negatively impacted the care recipients but also deteriorated the caregiving conditions among those who provide care [31, 33].

Stroke is one of major causes of disability, which leads to life-long impacts on both patients with stroke and their caregivers. Continuous caregiving for patients with stroke is a complex and multidimensional activity which involves handing physical, cognition, and communication impairments. This usually cumulates in negative health and social-related effects for caregivers of patients with stroke [34]. Factors related to the psychosocial stress of caregivers and the caring contexts for understanding caregiver burdens during the pandemic have been documented. However, little is known about how the pandemic has impacted on caregivers of stroke survivors. In addition, it remains unclear how satisfaction with support and fear of COVID-19 associate with caregiver burden when psychological distress and relevant demographics are controlled. Understanding the role of these factors in caregiver burden is significant during the pandemic as individuals experienced substantial psychological stress and fear related to the outbreak [35–37]. This study seeks to fill this knowledge gap by exploring how satisfaction with informal (family and friends) support, psychological stress, and fear of COVID-19 are associated with caregiver burdens among those who provide care to patients with stroke during the coronavirus pandemic.

## Methods

### Participants and procedure

This study uses a cross-sectional study design. The study participants were enrolled from E-Da Hospital, a medical center with approximately 1000 stroke admissions per year. All patients with stroke, including ischemic, hemorrhagic, and transient ischemic attack, received regular follow-up in the inpatient or outpatient department. Each patient's history was taken, and each received a neurological examination, brain image study, and stroke risk factors evaluation. For inclusion, caregivers over age 18 who cared for patients with stroke for more than 4 hours per day were enrolled. We used 4 hours per day as an inclusion criterion because caregivers who care for a patient with stroke more than 4 hours a day have significantly higher levels of stress than those who care less than 4 hours per day [38]. The caregivers needed to accompany the patients with stroke during acute-phase and regular outpatient follow-ups.

The exclusion criteria included (i) the caregiver cannot understand the questionnaire, (ii) the caregiver was confirmed to have dementia, other cognitive impairment, or severe hearing loss. Specifically, higher cortical and vestibulocochlear nerve examination were conducted for all patients with stroke after they agreed to participate in the study. For those with abnormal higher cortical functions, Mini-Mental State Examination and Clinical Dementia Rating were used to evaluate their cognitive functions. Regarding hearing function, vestibulocochlear nerve examination, including finger rubbing test, Weber and Rinne test were used.

The research assistants gave the caregivers verbal instructions and helped them complete the questionnaire during inpatient or outpatient follow-ups. Moreover, this study was approved by the institutional review board (IRB) of E-Da Hospital (Ref No. EMRP-110-079) and all the participants have signed a written informed consent before participation. Finally, there were 5 caregivers who did not answer the questionnaire. There were three other caregivers excluded because they could not speak Chinese.

### Measures

#### Caregiver burden

Caregiver burden was assessed with the 12-item Zarit Burden Interview (ZBI) developed by Ballesteros et al. [39] using a 5-point Likert scale (0=not at all; 4=extremely). A higher score in the 12-item ZBI indicates a higher level of caregiver burden. The Chinese 12-item ZBI has been validated and found to be equivalent to the original 22-item ZBI while outperforming the other short versions of ZBI [40, 41]. The current study averaged the 12 ZBI items to present the caregiver burden score. Cronbach’s α of the ZBI in the present sample was 0.97.

#### Satisfaction with support

Satisfaction with support was assessed from two sources: family and friends. Two items were used: "Are you satisfied with the support provided by your family in the past week?" and "Are you satisfied with the support provided by your friend in the past week?" Both items were rated on a 5-point Likert scale (1=strongly dissatisfied; 5= strongly satisfied), and a higher score indicates a higher level of satisfaction with support. The two items were retrieved from a previous study assessing satisfaction with support among general population in Taiwan [42]. Li et al. [42] originally designed three items to assess satisfaction with support (family members, friends, and colleagues/classmates). However, because patients with stroke are unlikely to have much interaction with their colleagues/classmates, we only adopted the two items on family members and friends. Moreover, Li et al. [42] showed that the three satisfaction with support items had good psychometric properties (Cronbach’s α = 0.81). Nevertheless, given that the two items indicate different types of satisfaction with support for the present sample, the two item scores were separately used.

#### Psychological distress

Psychological distress was assessed using the 21-item Depression, Anxiety, Stress Scale (DASS-21) developed by Lovibond and Lovibond [43]. The DASS-21 assessed psychological distress using a 4-point Likert scale (0=did not apply to me at all; 3=applied to me very much or most of the time). A higher score in the DASS-21 indicates a higher level of psychological distress. The Chinese DASS-21 has been validated and showed satisfactory psychometric properties [44]. The present study averaged the 21 DASS-21 items to present the psychological distress score. Cronbach’s α of the DASS-21 in the present sample was 0.85.

#### Fear of COVID-19

The 7-item Fear of COVID-19 Scale (FCV-19S) developed by Ahorsu et al. [45] was used to assess fear of COVID-19 using a 5-point Likert scale (1=strongly disagree; 5=strongly agree). A higher score in the FCV-19S indicates a higher level of fear of COVID-19. The Chinese FCV-19S has been validated and showed satisfactory psychometric properties [46–48]. The present study summed the 7 FCV-19S items to present the fear of COVID-19 score. Cronbach’s α of the FCV-19S in the present sample was 0.84.

#### Demographics and covariates

Participants' characteristics, such as age, sex, marital status, and educational years, were assessed using self-reports. Covariates of daily caregiving hours, whether as an informal caregiver or receiving caregiving assistance, were assessed using self-reports. Examples of the caregiving assistance in Taiwan included feeding, dressing, bathing, transferring patients from bed to wheelchair, moving patients from home to another place, and assisting patients when they are in daycare institute or receiving medical service [reference?].

### Data analysis

Descriptive statistics were used to examine the demographics and personal characteristics of the participants. Pearson correlations were then used to examine the bivariate correlations between the studied variables, including caregiver burden, satisfaction with family support, satisfaction with friend support, psychological distress, fear of COVID-19, daily caregiving hours, being an informal caregiver or not, and receiving assistance for caregiving. Finally, a multiple linear regression model was constructed to understand how satisfaction with support (including support from family and friends), psychological distress, and fear of COVID-19 explain caregiver burden. In the multiple linear regression model, age, sex (reference group: females), marital status (reference group: married), number of years of education, daily caregiving hour, informal caregiver or not (reference group: no), and receiving caregiving assistance (reference group: no) were controlled. All the statistical analyses were done using the IBM Corp. SPSS 20.0 (Armonk, NY).

## Results

A total of 171 caregivers were recruited for this study (Table 1). Most of the caregivers were informal caregivers (*n=*153; 89.5%) and females (*n=*113; 66.1%). The mean age of the participants was 53.73 (SD=11.77) years; their mean number of years of education was 10.98 (SD=4.11); and their mean caregiving hours per day was 17.80 (SD=8.82). Nearly 80% of the participants were currently married (*n=*136; 79.5%), and slighting over one-third of the participants did not have any assistance for their caregiving (*n=*61; 35.7%). On average, their score was 4.52 (SD=8.97) for caregiver burden; 4.39 (SD=0.80) for satisfaction with family support; 4.06 (SD=0.95) for satisfaction with friend support; 2.55 (SD=4.13) for psychological distress; and 8.44 (SD=3.50) for fear of COVID-19.

**Table 1:** Caregiver Characteristics (*N=*171)

	Mean ± SD	n (%)
Age, yr	53.73 ±11.77	
Sex		
Male		58 (33.9)
Female		113 (66.1)
Years of education	10.98 ± 4.11	
Daily hours of care	17.80 ± 8.82	
Marital status		
Married		136 (79.5)
Other		32 (18.7)
Missing		3 (1.8)
Assistance of caregiving		
No		61 (35.7)
Yes		110 (64.3)
Relationship with the patient		
Children		64 (37.4)
Spouse		65 (38.0)
Paid caregiver		15 (8.8)
Other		24 (14.0)
Missing		3 (1.8)
Caregiver burden	4.52 ± 8.97	
Satisfaction with family support	4.39 ± 0.80	
Satisfaction with friend support	4.06 ± 0.95	
Psychological distress	2.55 ± 4.13	
Fear of COVID-19	8.44 ± 3.50	

Table 2 demonstrates the bivariate correlations between the studied variables. More specifically, caregiver burden was negatively associated with satisfaction with family support (*r=*-0.19; *p=*0.02) and satisfaction with friend support (*r=*-0.18; *p=*0.03); and positively associated with psychological distress (*r=*0.30; *p<*0.001) and fear of COVID-19 (*r=*0.20; *p=*0.01).

**Table 2:** Correlation matrix using Pearson correlations for studied variables

				r (*p*-value)			
	1.	2.	3.	4.	5.	6.	7.
1. Daily caregiving hour	--						
2. Informal caregiver	0.41 (<0.001)	--					
3. Assistance	-0.21 (0.01)	-0.24 (0.002)	--				
4. Family satisfaction	-0.09 (0.27)	-0.02 (0.78)	-0.07 (0.39)	--			
5. Friend satisfaction	-0.23 (0.006)	-0.14 (0.08)	-0.12 (0.14)	0.67 (<0.001)	--		
6. Psychological distress	0.04 (0.63)	-0.03 (0.67)	-0.03 (0.73)	-0.270 (<0.001)	-0.31 (<0.001)	--	
7. Fear of COVID-19	0.14 (0.11)	-0.06 (0.49)	0.08 (0.29)	-0.02 (0.80)	0.03 (0.73)	0.26 (0.001)	--
8. Caregiver burden	0.16 (0.054)	0.12 (0.12)	0.01 (0.91)	-0.19 (0.02)	-0.18 (0.03)	0.30 (<0.001)	0.20 (0.01)

*Assistance* Assistance with caregiving, *Family satisfaction* Satisfaction with family support, *Friend satisfaction* Satisfaction with friend support

The multiple linear regression model (Table 3) with the adjustments of confounders (i.e., age, sex, marital status, number of years of education, daily caregiving hours, informal caregiver or not, having caregiving assistance or not) showed that the entire model is significant (F-value=3.23; *p=*0.001; R^2^=0.24; adjusted R^2^=0.16). Moreover, caregiver burden was negatively associated with satisfaction with family support (standardized coefficient=-0.24; *p=*0.03) but not satisfaction with friend support (standardized coefficient=0.06; *p=*0.62). Caregiver burden was positively associated with psychological distress (standardized coefficient=0.20; *p=*0.047) and fear of COVID-19 (standardized coefficient=0.23; *p=*0.01).

**Table 3:** Linear regression models explaining caregiver burden

**Dependent variable**	**Unstand. Coeff. (SE)**	**Stand. Coeff.**	***P*-value**
Age (year)	0.04 (0.10)	0.05	0.68
Sex (Ref: female)	-0.20 (1.91)	-0.01	0.92
Marital status (Ref: married)	-0.16 (2.27)	-0.01	0.94
Educational year	0.40 (0.25)	0.17	0.12
Daily caregiving hours	0.13 (0.11)	0.12	0.24
Informal caregiver (Ref: no)	4.52 (3.01)	0.14	0.14
Assistance of caregiving (Ref: no)	1.44 (1.76)	0.07	0.42
Satisfaction of family support	-2.92 (1.35)	-0.24	0.03*
Satisfaction of friend support	0.54 (1.11)	0.06	0.62
Psychological distress	0.47 (0.23)	0.20	0.047*
Fear of COVID-19	0.67 (0.26)	0.23	0.01*

R^2^=0.24; Adj. R^2^=0.16; entire model *p*-value=0.001

*Unstand. Coeff* Unstandardized coefficient, *SE* Standard error, *Stand. Coeff* Standardized coefficient

^*^p < 0.05

## Discussion

In this study, we found that during the COVID-19 pandemic, the caregiver burdens of caregivers who provided care to patients with stroke were relatively minor, compared to other previous studies on patients with stroke conducted before the pandemic (ZBI mean score standardized using 0-4 scale in the present study = 0.38; ZBI mean score standardized using 0-4 scale = 1.22 [49]; ZBI mean score standardized using 0-4 scale = 1.35 [50]; ZBI mean score standardized using 0-4 scale = 2.28 [51]). The fear of COVID-19 was relatively low in the present sample, and the low levels of caregiver burden and fear of COVID-19 could be due to the relatively mild COVID-19 outbreak in Taiwan [48, 52, 53]. Moreover, family support seemed to relieve the burdens of providing care to patients with stroke. In contrast, psychological stress and fear of the pandemic could be positively associated with higher caregiver burdens.

Previous research has identified that caregivers who experience negative psychological symptoms, such as depression and anxiety, tend to have higher caregiver burdens [54–56]. This study obtained consistent results on the positive correlations between caregivers' psychological stress and their caregiving-related burdens and applied this finding to the conditions during the pandemic. Also, this study covered a specific factor related to caregivers' emotions during the pandemic. It demonstrated that increased fear of COVID-19 may correlate positively with higher caregiver burdens among those who provide care to patients with stroke.

The identification of the relationship between the fear of COVID-19 and caregiver burdens is substantial in two aspects. First, although the fear of COVID-19 is related to psychological stress, their relationships are highly heterogeneous [57]. Specifically, fear is usually considered as a trigger for people to develop coping strategies (e.g., fight or flight) to respond to the unknown situations. When people cannot control their fear, they are likely to develop different types of psychological distress, including caregiver burden. Second, psychological distress assessed in the present study was not specifically focused on COVID-19. Therefore, it could be “COVID-19” contributing to a large proportion of variance in caregiver burden. Subsequently, fear of COVID-19 could be a stronger factor than the psychological distress to explain caregiver burden in the present study. However, it is noted that the fear of COVID-19 could be highly associated with a wide range of psychological problems [58]. Thus, further investigations are encouraged to examine whether the fear of COVID-19 could be an independent explanatory factor or acts through the proxy of mental health-related variables.

This study explored the role of informal support in caregiver burdens during the pandemic and found an interesting contrast between family support and wider social support, such as that from friends and neighbors. In Taiwan as well as in other East Asian contexts, influenced by prevailing Confucian cultural values that emphasizes family’s caring obligations and limited public care services development, caregiving for disabled people largely relies on kinship networks, including spouses, children, and relatives [59]. The results demonstrated that satisfaction with family support might relieve caregiver burden during the pandemic, whereas caregivers' perceived satisfaction with friend support did not have similar effects. These differences between family and wider social support on caregiver burdens illuminate the mechanisms of burden relieving effects. Furthermore, some research revealed that perceived social support has a stronger predictive effect on caregiver burden than received social support [60]. In the case of the pandemic, it is unclear whether higher caregiver burdens were caused by insufficient and/or inadequate care support when informal support failed to compensate for the limited availability of formal services or resulted from psychosocial expectations about informal support when caregivers were in need.

The research findings also bring an implication for health care professionals. As mentioned above, informal caregivers, especially family members, play the dominant role in caregiving for patients with stroke in Taiwan [59], whereas health care professionals provide relatively limited long-term care services. This somewhat explains why our findings indicated that over half of the caregiver for patients with stroke were a family member of the patient (either spouse or children). Health care providers focus more on acute medical management and rehabilitation programs at acute care settings. By Contrast, support for caregivers, such as caregiving training and home-based services, is significantly limited.

The research findings may suggest that through caregiver support program development healthcare providers can help relieve the caregiver burden on caregivers by reducing their psychological distress and fear of COVID-19. Healthcare providers may also consider mobilize family support for these caregivers to reduce their caregiver burden.

The present study has some limitations. First, this study was a cross-sectional research design, and therefore clarifying the causal relationship between caregiver burden and psychosocial characteristics of caregivers requires further longitudinal study designs. In addition, the cross-sectional analysis of caregiver burdens and associated factors limited its potential to evaluate the change in caregiver burdens and risk factors for higher caregiver burdens before and during the pandemic. A third limitation relates to the generalization of this study. The adoption of convenience sampling limits the representativeness of the present sample and the potential to generalize the results. In particular, this research was conducted in Taiwan, where kinship networks play a significant role in caregiving support. This characteristic may limit the generalization of the research findings, while the role of caregiving support varies across countries. Fourth, we did not assess the condition of the patients with stroke (e.g. the limitation of their daily activities; their dependency level of activities of daily living). Such condition may impact on the caregiver burden and serve a confounder to the present study’s findings. Lastly, length of caregivers’ experience to care people with chronic illness is usually an important factor explaining caregiver burden. However, we did not assess such information and the present findings may be confounded by the length of caregivers’ experience in caring.

## Conclusion

In conclusion, caregiver burden is an issue requiring attention during the COVID-19 pandemic. The physical and mental health of care recipients and caregivers has deteriorated due to restrictive measures, and the limited availability and accessibility of formal health and care support have negatively impacted those in need. Lower satisfaction with family support, higher levels of psychological distress, and greater fear of COVID-19 were associated with increased caregiver burdens among caregivers of patients with stroke. This finding suggests that during a period of decreased social connectedness and limited traditional formal care services, we need to mobilize and provide sufficient support to those who provide unpaid long-term care to their family.

## Data Availability

The data that support the findings of this study are available from the corresponding author upon reasonable request.

## Abbreviations

ZBI: Zarit Burden Interview
DASS-21: Depression, Anxeity, Stress Scale
FCV-19S: Fear of COVID-19 Scale

## References

[1] Hasannia E, Mohammadzadeh F, Tavakolizadeh M, Davoudian N, Bay M (2021). Assessment of the anxiety level and trust in information resources among iranian health-care workers during the pandemic of coronavirus disease 2019. Asian J Soc Health Behav.

[2] Liu E, Arledge S (2022). Individual characteristics and demographics associated with mask wearing during the COVID-19 pandemic in the United States. Asian J Soc Health Behav.

[3] Olashore AA, Akanni OO, Fela-Thomas AL, Khutsafalo K (2021). The psychological impact of COVID-19 on health-care workers in African Countries: A systematic review. Asian J Soc Health Behav.

[4] Patel BR, Khanpara BG, Mehta PI, Patel KD, Marvania NP (2021). Evaluation of perceived social stigma and burnout, among health-care workers working in covid-19 designated hospital of India: a cross-sectional study. Asian J Soc Health Behav.

[5] Prasiska DI, Muhlis ANA, Megatsari H (2022). Effectiveness of the emergency public activity restrictions on COVID-19 epidemiological parameter in East Java Province, Indonesia: An ecological study. Asian J Soc Health Behav.

[6] Rad MK, Fakhri A, Stein L, Araban M (2022). Health-care staff beliefs and coronavirus disease 2019 vaccinations: a cross-sectional study from Iran. Asian J Soc Health Behav.

[7] Rajabimajd N, Alimoradi Z, Griffiths MD (2021). Impact of COVID-19-related fear and anxiety on job attributes: A systematic review. Asian J Soc Health Behav.

[8] Shirali GA, Rahimi Z, Araban M, Mohammadi MJ, Cheraghian B (2021). Social-distancing compliance among pedestrians in Ahvaz, South-West Iran during the Covid-19 pandemic. Asian J Soc Health Behav.

[9] Piroth L, Cottenet J, Mariet A-S, Bonniaud P, Blot M, Tubert-Bitter P, Quantin C (2021). Comparison of the characteristics, morbidity, and mortality of COVID-19 and seasonal influenza: a nationwide, population-based retrospective cohort study. Lancet Respir Med.

[10] ACAPS: COVID-19 Government Measures Dataset. In.; 2021. [cited 2022 May 3]. Available from: https://www.acaps.org/covid-19-government-measures-dataset.

[11] Zylke JW, Bauchner H (2020). Mortality and morbidity: the measure of a pandemic. JAMA.

[12] Bailey L, Ward M, DiCosimo A, Baunta S, Cunningham C, Romero-Ortuno R, Kenny RA, Purcell R, Lannon R, McCarroll K (2021). Physical and mental health of older people while cocooning during the COVID-19 pandemic. QJM.

[13] Williams R, Jenkins DA, Ashcroft DM, Brown B, Campbell S, Carr MJ, Cheraghi-Sohi S, Kapur N, Thomas O, Webb RT (2020). Diagnosis of physical and mental health conditions in primary care during the COVID-19 pandemic: a retrospective cohort study. Lancet Public Health.

[14] Moynihan R, Sanders S, Michaleff ZA, Scott AM, Clark J, To EJ, Jones M, Kitchener E, Fox M, Johansson M (2021). Impact of COVID-19 pandemic on utilisation of healthcare services: a systematic review. BMJ Open.

[15] Shippee TP, Akosionu O, Ng W, Woodhouse M, Duan Y, Thao MS, Bowblis JR (2020). COVID-19 pandemic: exacerbating racial/ethnic disparities in long-term services and supports. J Aging Soc Policy.

[16] WHO: The impact of COVID-19 on health and care workers: a closer look at deaths. In.: World Health Organization; 2021. [cited 3 May 2022]. Available from: https://apps.who.int/iris/bitstream/handle/10665/345300/WHO-HWF-WorkingPaper-2021.1-eng.pdf?sequence=1&isAllowed=y.

[17] Fong TK-H, Cheung T, Chan W-C, Cheng CP-W: Depression, anxiety and stress on caregivers of persons with dementia (CGPWD) in Hong Kong amid COVID-19 pandemic. Int J Environ Res Public Health. 2021, 19(1):184. 10.3390/ijerph19010184 PMC8751129 35010451

[18] Garcia-Rudolph A, Sauri J, Garcia-Molina A, Cegarra B, Opisso E, Tormos JM, Frey D, Madai VI, Bernabeu M (2022). The impact of coronavirus disease 2019 on emotional and behavioral stress of informal family caregivers of individuals with stroke or traumatic brain injury at chronic phase living in a Mediterranean setting. Brain Behav.

[19] Irani E, Niyomyart A, Zauszniewski JA (2021). Caregiving stress and self-rated health during the COVID-19 pandemic: The mediating role of resourcefulness. Issues Ment Health Nurs.

[20] Bergmann M, Wagner M: The impact of COVID-19 on informal caregiving and care receiving across Europe during the first phase of the pandemic. Front Public Health. 2021;9:673874. 10.3389/fpubh.2021.673874 PMC8242257 34222177

[21] Cohen SA, Nash CC, Greaney ML (2021). Informal Caregiving during the COVID-19 Pandemic in the US: Background, Challenges, and Opportunities. Am J Health Promot.

[22] Rodrigues R, Simmons C, Schmidt AE, Steiber N (2021). Care in times of COVID-19: The impact of the pandemic on informal caregiving in Austria. Eur J Ageing.

[23] Budnick A, Hering C, Eggert S, Teubner C, Suhr R, Kuhlmey A, Gellert P (2021). Informal caregivers during the COVID-19 pandemic perceive additional burden: findings from an ad-hoc survey in Germany. BMC Health Serv Res.

[24] Cohen SA, Kunicki ZJ, Drohan MM, Greaney ML (2021). Exploring changes in caregiver burden and caregiving intensity due to COVID-19. Gerontol Geriatr Med.

[25] Lightfoot E, Yun H, Moone R, Otis J, Suleiman K, Turck K, Kutzler C (2021). Changes to family caregiving of older adults and adults with disabilities during COVID-19. Gerontol Geriatr Med.

[26] Seo HJ, Kim SM, Park JM (2021). Impact of the COVID-19 pandemic on the caregiver burden and quality of life in caregivers of patients with dementia. Alzheimer’s Dement.

[27] Kazemi A, Azimian J, Mafi M, Allen K-A, Motalebi SA (2021). Caregiver burden and coping strategies in caregivers of older patients with stroke. BMC Psychol.

[28] Borges-Machado F, Barros D, Ribeiro Ó, Carvalho J (2020). The effects of COVID-19 home confinement in dementia care: physical and cognitive decline, severe neuropsychiatric symptoms and increased caregiving burden. Am J Alzheimers Dis Other Demen.

[29] Kazawa K, Kubo T, Ohge H, Akishita M, Ishii S (2021). Preparedness guide for people with dementia and caregivers in COVID-19 pandemic. Geriatr Gerontol Int.

[30] Tsapanou A, Papatriantafyllou JD, Yiannopoulou K, Sali D, Kalligerou F, Ntanasi E, Zoi P, Margioti E, Kamtsadeli V, Hatzopoulou M (2021). The impact of COVID-19 pandemic on people with mild cognitive impairment/dementia and on their caregivers. Int J Geriatr Psychiatry.

[31] Leira EC, Russman AN, Biller J, Brown DL, Bushnell CD, Caso V, Chamorro A, Creutzfeldt CJ, Cruz-Flores S, Elkind MS (2020). Preserving stroke care during the COVID-19 pandemic: potential issues and solutions. Neurology.

[32] Otobe Y, Kimura Y, Suzuki M, Koyama S, Kojima I, Yamada M (2022). Factors Associated with Increased Caregiver Burden of Informal Caregivers during the COVID-19 Pandemic in Japan. J Nutr Health Aging.

[33] Sutter-Leve R, Passint E, Ness D, Rindflesch A (2021). The caregiver experience after stroke in a covid-19 environment: A qualitative study in inpatient rehabilitation. J Neurol Phys Ther.

[34] Camak DJ (2015). Addressing the burden of stroke caregivers: a literature review. J Clin Nurs.

[35] Ahorsu DK, Lin C-Y, Pakpour AH (2020). The association between health status and insomnia, mental health, and preventive behaviors: the mediating role of fear of COVID-19. Gerontol Geriatr Med.

[36] Wang C, Pan R, Wan X, Tan Y, Xu L, Ho CS, Ho RC (2020). Immediate psychological responses and associated factors during the initial stage of the 2019 coronavirus disease (COVID-19) epidemic among the general population in China. Int J Environ Res Public Health.

[37] Xiang Y-T, Yang Y, Li W, Zhang L, Zhang Q, Cheung T, Ng CH (2020). Timely mental health care for the 2019 novel coronavirus outbreak is urgently needed. Lancet Psychiatry.

[38] Bhattacharjee M, Vairale J, Gawali K, Dalal PM (2012). Factors affecting burden on caregivers of stroke survivors: Population-based study in Mumbai (India). Ann Indian Acad Neurol.

[39] Ballesteros J, Santos B, González-Fraile E, Muñoz-Hermoso P, Domínguez-Panchón AI, Martín-Carrasco M (2012). Unidimensional 12-item Zarit Caregiver Burden Interview for the assessment of dementia caregivers’ burden obtained by item response theory. Value Health.

[40] Lin C-Y, Wang J-D, Pai M-C, Ku L-JE: Measuring burden in dementia caregivers: confirmatory factor analysis for short forms of the Zarit Burden Interview. Arch Gerontol Geriatr. 2017;68:8–13. 10.1016/j.archger.2016.08.005. 10.1016/j.archger.2016.08.005 27580015

[41] Lin C-Y, Ku L-JE, Pakpour AH: Measurement invariance across educational levels and gender in 12-item Zarit Burden Interview (ZBI) on caregivers of people with dementia. Int Psychogeriatr. 2017, 29(11):1841–1848. 10.1017/S1041610217001417. 10.1017/S1041610217001417 28760167

[42] Li D-J, Ko N-Y, Chen Y-L, Wang P-W, Chang Y-P, Yen C-F, Lu W-H (2020). COVID-19-related factors associated with sleep disturbance and suicidal thoughts among the Taiwanese public: a Facebook survey. Int J Environ Res Public Health.

[43] Lovibond SH, Lovibond PF: Manual for the depression anxiety stress scales. Australia: Psychology Foundation; 1996.

[44] Wang K, Shi H-S, Geng F-L, Zou L-Q, Tan S-P, Wang Y, Neumann DL, Shum DH, Chan RC (2016). Cross-cultural validation of the depression anxiety stress scale–21 in China. Psychol Assess.

[45] Ahorsu DK, Lin C-Y, Imani V, Saffari M, Griffiths MD, Pakpour AH: The fear of COVID-19 scale: development and initial validation. Int J Ment Health Addict. 2020; 1–9. 10.1007/s11469-020-00270-8. 10.1007/s11469-020-00270-8 PMC7100496 32226353

[46] Chang K-C, Hou W-L, Pakpour AH, Lin C-Y, Griffiths MD (2022). Psychometric testing of three COVID-19-related scales among people with mental illness. Int J Ment Health Addict.

[47] Chen I-H, Chen C-Y, Zhao K-Y, Gamble JH, Lin C-Y, Griffiths MD, Pakpour AH: Psychometric evaluation of fear of COVID-19 Scale (FCV-19S) among Chinese primary and middle schoolteachers, and their students. Curr Psychol. 2022;1–17. 10.1007/s12144-021-02471-3. 10.1007/s12144-021-02471-3 PMC8727075 35002189

[48] Lin CY, Hou WL, Mamun MA, Aparecido da Silva J, Broche‐Pérez Y, Ullah I, Masuyama A, Wakashima K, Mailliez M, Carre A: Fear of COVID-19 Scale (FCV‐19S) across countries: Measurement invariance issues. Nurs Open. 2021;8(4):1892–1908. 10.1002/nop2.855. 10.1002/nop2.855 PMC8186712 33745219

[49] Imarhiagbe FA, Asemota A, Oripelaye B, Akpekpe J, Owolabi A, Abidakun A, Akemokwe F, Ogundare V, Azeez A, Osakue J: Burden of informal caregivers of stroke survivors: validation of the Zarit burden interview in an African population. Ann Afr Med. 2017 Apr-Jun;16(2):46–51. doi: 10.4103/aam.aam_213_16. 10.4103/aam.aam_213_16 PMC5452708 28469116

[50] Caro CC, Costa JD, Da Cruz DMC (2018). Burden and quality of life of family caregivers of stroke patients. Occup Ther Health Care.

[51] Long NX, Pinyopasakul W, Pongthavornkamol K, Panitrat R (2019). Factors predicting the health status of caregivers of stroke survivors: A cross-sectional study. Nurs Health Sci.

[52] Kuo Y-J, Chen Y-P, Wang H-W, Liu C-h, Strong C, Saffari M, Ko N-Y, Lin C-Y, Griffiths MD: Community outbreak moderates the association between COVID-19-related behaviors and COVID-19 fear among older people: a one-year longitudinal study in Taiwan. Front Med (Lausanne). 2021;8:756985. 10.3389/fmed.2021.756985. 10.3389/fmed.2021.756985 PMC8719620 34977064

[53] Pakpour AH, Liu C-h, Hou W-L, Chen Y-P, Li Y-P, Kuo Y-J, Lin C-Y, Scarf D: Comparing fear of COVID-19 and preventive COVID-19 infection behaviors between Iranian and Taiwanese older people: early reaction may be a key. Front Public Health. 2021;9:740333. 10.3389/fpubh.2021.740333. 10.3389/fpubh.2021.740333 PMC8495067 34631652

[54] Beach SR, Schulz R, Williamson GM, Miller LS, Weiner MF, Lance CE (2005). Risk factors for potentially harmful informal caregiver behavior. J Am Geriatr Soc.

[55] Connors MH, Seeher K, Teixeira-Pinto A, Woodward M, Ames D, Brodaty H (2020). Dementia and caregiver burden: A three-year longitudinal study. Int J Geriatr Psychiatry.

[56] Grov EK, Fosså SD, Sørebø Ø, Dahl AA (2006). Primary caregivers of cancer patients in the palliative phase: a path analysis of variables influencing their burden. Soc Sci Med.

[57] Erbiçer ES, Metin A, Çetinkaya A, Şen S: The relationship between fear of COVID-19 and depression, anxiety, and stress. European Psychologist, 26(4), 323–333. 10.1027/1016-9040/a000464.

[58] Şimşir Z, Koç H, Seki T, Griffiths MD (2022). The relationship between fear of COVID-19 and mental health problems: A meta-analysis. Death Stud.

[59] Yeh M-J: Long-term care system in Taiwan: the 2017 major reform and its challenges. Ageing and Society, 40(6), 1334–1351. 10.1017/S0144686X18001745.

[60] Chiou CJ, Chang H-Y, Chen IP, Wang HH (2009). Social support and caregiving circumstances as predictors of caregiver burden in Taiwan. Arch Gerontol Geriatr.

